# Harmonisation and standardisation of health sector and programme reviews and evaluations – how can they better inform health policy dialogue?

**DOI:** 10.1186/s12961-016-0161-9

**Published:** 2016-12-16

**Authors:** Juliet Nabyonga-Orem, Prosper Tumusiime, Jennifer Nyoni, Aku Kwamie

**Affiliations:** 1 Health Systems and Services Cluster, Health Systems Governance, World Health Organization, Inter-Country Support Team for Eastern & Southern Africa, P.O. Box CY 348, Causeway, Harare, Zimbabwe; 2 Health Systems and Services cluster, World Health Organization Regional office for Africa, BP 06, Cite de Djoue, Brazzaville, Congo; 3 Ghana Health Service, Research and Development Division, PMB M9, Ministries, Accra, Ghana

**Keywords:** Health sector, Health programmes, Performance assessment, Use of evidence, Policy dialogue

## Abstract

**Background:**

Health sector and programme performance assessments provide a rich source of contextual data directly linked to implementation of programmes and can inform health policy dialogue, planning and resource allocation. In seeking to maximise this opportunity, there are challenges to overcome. A meeting convened by the World Health Organization African Region discussed the strengths, weaknesses and challenges to harmonising and standardising health sector and programme performance assessments, as well as use of evidence from such processes in decision making. This article synthesises the deliberations which emerged from the meeting. Discussing these in light of other literature we propose practical options to standardising health sector and programme performance assessment and improve realisation of using evidence in decision making.

**Discussion:**

Use of evidence generated from health sector and programme performance assessments into regular country processes of sectoral monitoring, dialogue and policy modification is crucial. However, this process faces several challenges. Identified challenges were categorised under several themes, namely the weak institutional capacities for monitoring and evaluation in reference to weak health information systems, a lack of tools and skills, and weak accountability mechanisms; desynchronised planning timeframes between programme and overall health sector strategies; inadequate time to undertake comprehensive and good quality performance assessment; weak mechanisms for following up on implementation of recommendations; lack of effective stakeholder participation; and divergent political aspirations.

**Conclusion:**

The question of what performance assessment is for in a country must be asked and answered clearly if the utility of these processes is to be realised. Standardising programme and sector reviews offers numerable opportunities that need to be maximised. Identified challenges need to be overcome through strengthened Ministry of Health leadership, effective stakeholder engagement and institutionalising follow-up mechanisms for agreed recommendations. In addition, health sector performance assessments need to be institutionalised as part of the accountability mechanism, and they must be planned for and funding secured within annual budget and medium term expenditure frameworks.

## Background

Health sector and programme performance assessments are a rich source of contextual data directly linked to the implementation of country strategic and operational plans. Ideally, these assessments should be a good source of evidence to inform decision making, planning and resource allocation at sub-national and national levels. They should also inform stakeholder dialogue through joint-review processes surrounding decision making about allocation and distribution of human and financial resources, infrastructure, medicines and technologies. Stakeholders are herein defined “*as actors who have an interest in the issue under consideration, who are affected by the issue or, who – because of their positions – have or could have an active or passive influence on the decision making or implementation processes*” [1]. We adopt the definition of performance assessment provided by Smith et al. [2] as “*seeking to monitor, evaluate, and communicate the extent to which key objectives are met*”. The process involves collection, review and use of information for a purpose [3]. Health sector and programme performance assessments use data from several sources, including the health management information system, service availability and readiness assessment, administrative data, national health accounts, and surveys such as the demographic health surveys and household living surveys.

The Sixty Fourth World Health Assembly urges member states to “*regularly monitor, review and adjust their national or subnational health policies, strategies and plans with a view to developing evidence-based responses to evolving challenges and opportunities, and to involve all relevant stakeholders*” [4]. This is intended to lay a foundation of two prerequisite actions, namely (1) the regular involvement of stakeholders in performance assessment and (2) the use of evidence in decision-making, both of which call for engagement of health actors in policy dialogue. In this article, we adopt the definition of health policy dialogue by Dheepa et al. [5], as “*a dialogue that is part and parcel of the policy and decision-making processes, intended to contribute to developing or implementing a policy change following a round of evidence-based discussions/workshops/consultations on a particular subject*”.

Anecdotal data from country actors indicates multiple challenges to maximising the relevancy of performance assessments. These include issues of data quality and timeliness from routine health information systems, tensions in the generation of bottom-up evidence and the propagation of top-down priorities, and conflicting planning and resource allocation timeframes between the sector and programmes. While there are reports detailing lessons from strengthening in-country monitoring and evaluation (M&E) systems [6–8], the bulk of these are written from donor perspectives; the literature is near-void of documented perspectives from countries themselves.

Countries develop national health policies and medium-term (i.e. 3–5 year) sector strategic plans for operationalising policy aspirations. Strategic plans are implemented through annual operation plans which are meant to be aligned to annual budget cycles. Performance assessment of such plans needs to be undertaken regularly against set indicators to assess attainment of progress and modify the input of resources for improved service delivery as necessary. M&E plans are developed to guide health sector performance assessment. Results emanating from such assessments need to improve evidence-based planning and decision-making, as well as overall health sector performance. M&E plans detail the indicators for performance assessment, data sources to be used, periodicity of reporting, institutional strengthening activities, roles of the different actors and how results will inform decision-making [9]. Vertical programmes (such as malaria, tuberculosis, and immunisation) develop strategic and operational plans as well. Such programmatic strategies should be developed in line with overall sector objectives and strategies and with synchronised implementation timeframes. In terms of M&E, core programmatic indicators need to be included in the overall M&E plans. While programmes may maintain their own indicators for more comprehensive programme assessment, their core indicators must be aligned with the assessment of the health sector strategic plan and feed into the overall health sector performance assessment. In this article, we use the term performance assessment to denote annual reviews and evaluation of strategic plans.

Currently in the WHO African Region, all the 47 member states have national health strategic plans in place, and most, 38 out of the 47 countries, have institutionalised annual health sector reviews and undertake regular evaluation of their strategic plans. A meeting convened by the WHO Africa Regional Office in December 2015 brought together M&E country teams (comprised of head of M&E in the Ministry of Health (MoH) (n = 2 per country) and M&E technical officers from the WHO country office (n = 1 per country)) and senior malaria, AIDS, tuberculosis, reproductive, maternal, newborn, child and adolescent health, and nutrition technical officers from WHO. The 36 meeting participants were from ten countries (Ethiopia, Ghana, Kenya, Lesotho, Liberia, Malawi, The Gambia, Uganda, Zambia and Zimbabwe). The 10 countries were selected based on language (Anglophone, given the fact that the meeting would be conducted in English) and with the prerequisite of institutionalised annual health sector reviews and evaluation of strategic plans. The only country that did not take part was Tanzania due to government restrictions in missions outside the country; however, there were no anticipated differences between Tanzania and the countries that participated. Tanzania, like the other countries, has had periodic health sector reviews done annually. There was a general spread over the 10 countries represented. Although it had been initially intended to have participation both from the WHO country office and the MoH, Lesotho, Zambia and Zimbabwe did not have the MoH representatives while Kenya had only Ministry and County health officials represented. The persons from the MoHs were nationals of those particular countries and to some extent a good number of the WHO Country Office representatives.

As far as we are aware, this was the first meeting in the WHO African Region to be convened with the objective of reviewing and making recommendations for a harmonised and standardised approach to health sector and programme reviews and evaluations in order to realise a comprehensive health sector performance assessment, as well as use of evidence in policy dialogue and decision making. It is expected that there would be opportunity for holding such meetings periodically to learn and improve on undertaking and making use of the sector reviews. A report of the meeting was produced and the follow-up actions include using the outcome of the meeting to inform the development of a harmonised guide for health sector reviews.

Use of evidence in policy development and decision-making has gained prominence over the last decade. Scholars have highlighted facilitating factors as well as barriers to uptake of evidence. Among the facilitating factors is the quality and timeliness of the evidence, credibility of researches, providing practical solutions and dissemination in digestible forms [10]. However, we note that much of this literature refers to evidence derived from systematic research processes as opposed to evidence from routine information systems like health management information systems. Policymakers in low-income countries have pointed out the fact that the different types of evidence, both from formal (systematic research processes and routine information systems) and informal systems like community complaints do play a role on policy development [11–13].

Lavis et al. [10] cite four approaches that can be employed, either singly or in combination, to link research to action, namely the push efforts by researchers, the pull efforts by users, the exchange efforts with partnership between researchers and users, and use of large-scale knowledge translation platforms. The health sector reviews of health sector strategic plans in the African Region have mainly tried to harness information to respond to issues pertaining to the performance of the sector towards agreed targets. Although they can profit from the four approaches, they have commonly used pull efforts by using information either from appropriate research or from other data sources to influence action. Attempts at exchange efforts have performed suboptimally due to the limited participation of researchers in sector reviews, the large numbers of which are not conducive for effective dialogue and varied skills among the actors involved [14].

This debate article outlines the deliberations that emerged from the meeting, identifying the strengths, weaknesses and challenges to harmonising and standardising health sector and programme performance assessment. By synthesising these discussions and country and programme experiences, we seek to document and provide evidence to support the strengthening of the health sector and programme performance assessments and as well as country-level M&E, evidenced-based planning and policy modification. We conclude by proposing practical options to improving the use of evidence from performance assessments in health policy dialogue and decision-making at all levels of the health system. The proposed practical solution will further be incorporated in a guide to “Harmonize and Standardize Health Sector Reviews and Evaluations in the WHO African Region”, which is currently under development.

## Findings

### Obstacles to relevant performance assessments

The meeting identified several issues as challenges that currently prevent the full realisation of health sector and programme assessment utility in country-level planning cycles. The main themes that emerged were (1) weak institutional capacities for M&E; (2) desynchronised planning timeframes between programmes and overall health sector strategies; (3) inadequate time to undertake comprehensive and good quality performance assessments; (4) weak mechanisms for follow-up; (5) lack of effective stakeholder participation; and (6) divergent political aspirations. We take each issue in turn.

### Weak institutional capacities for M&E

Ensuring good quality evidence from performance assessments calls for adequate institutional capacity for M&E in terms of strong national health information systems, availability of tools and skills. Meeting participants noted that programme M&E activities were regularly better-resourced and had stronger M&E capacity. Participants partly attributed this to the increased funding from global health initiatives (GHIs) and other donor funds, and the accompanying stringent reporting requirements. Although GHIs have invested in health system strengthening and M&E, available evidence showed that investments were made in specific programme areas rather than within the health system generally [15]. GHIs have always emphasised that it is the responsibility of national authorities to identify general areas of support [16]. Related to this was the issue of resources for running annual reviews themselves; one country in particular noted that the lack of funds prevented them from holding annual reviews for 2 years. In several of the countries present participants shared that performance assessments were funded by donors with insignificant financial inputs from national governments.

Most countries have significant funding from donors amounting to approximately 40%, especially for programme operations [17]. However, the data are government data that would be available and hence should not constitute a big barrier. Although, in cases where the data is handled and kept by the organisations, data that may not be in favour of the organisations’ objectives will not be easy to access. Reference to donors in this paper is for the usual bilateral and multilateral donors in most of the countries such as United Nations agencies, United States Agency for International Development, UK Department for International Development, Swedish International Development Cooperation Agency, The Global Fund against AIDS, Tuberculosis and Malaria, and PEPFAR, among others.

Performance assessments are routine processes that must be planned for and funding secured within annual budgeting processes. The gaps in planning for these processes may partly explain the inferior quality reported in some countries as well as omissions where annual reviews are missed. This presents a missed opportunity for evidence-based dialogue and resource allocation. Paradoxically, while gaps in data are a problem, parallels in data collection systems and the multiplicity of data collection tools and reporting channels is a longstanding problem, which in turn compromise data quality.

Although performance assessments should serve as a mechanism of mutual accountability, weak ownership and limited participation of senior level MoH officers was a matter of concern. This lack of ownership also translates to policy dialogue processes where, often, the MoH should be seen as the lead organisation for vision and coordination, but is not. Such a state of affairs runs contrary to the principles of the Paris Declaration, which emphasise country ownership and mutual accountability [18].

### Desynchronised planning timeframes between programmes and overall health sector strategies

**Fig. 1:**
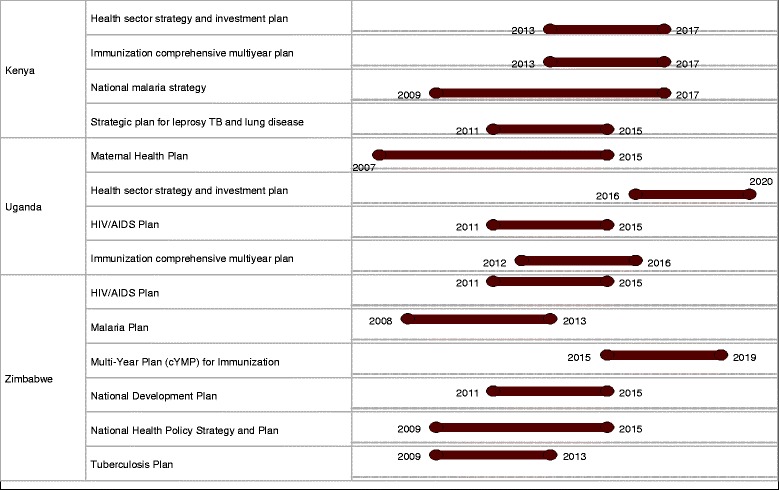
Planning cycles for health sector and programme strategic plans. Source: WHO country planning cycle database: http://www.nationalplanningcycles.org/planning-cycle/. Accessed 25 May 2015

Ensuring that programme performance assessment informs overall sector performance calls for synchronisation at the planning stage with regards to timeframes of the strategic and operation plans. In reality, this is not yet the case in many of the countries. Figure 1 depicts the overlap and desynchronisation of programme planning cycles in Kenya, Uganda and Zimbabwe as an illustration. There is no alignment, with reference to timeframes, between programme strategic plans and the national health sector strategic plan. In many countries, the same actors will be involved across programmes creating burdens of time and priority. In such instances, reviews and performance assessments are done at different times of the year resulting in multiplicity of review fora which participants noted as a hindrance to full stakeholder participation. Suboptimal discussions given the limited time to internalise the evidence, and contradictory decisions given the lack of a comprehensive view of sector performance also emerge as challenges. Additionally, the multiplicity of meeting fora stretches the already limited M&E capacities. This desynchronisation does not allow for alignment of reviews and realising a comprehensive health sector performance assessment.

### Inadequate time to undertake comprehensive and good quality performance assessments

Another issue with timing relates to the time required to actually undertake annual reviews and evaluations. This was noted to vary in the different countries between 2 and 4 months in the case of annual reviews and 3 and 6 months in the case of evaluations of health sector strategies. Longer periods impact on the ability to implement health programmes as staff are drawn away into undertaking assessments. The issue of undertaking subnational reviews and pooling findings into the overall review, coupled with the quest to ensure quality, calls for adequate time.

Use of evidence in decision making is impacted by the quality of the evidence [4, 5]. The quality of the reviews varied considerably and some attributed this to the methodologies employed, capacity of the teams involved and type of data used. Use of peer learning, looking at comparable subnational entities as well as countries, was discussed as one the ways to improve performance. This is difficult given the different methodologies employed. On a positive note, however, was the case of Ghana, where a standardised review guideline was provided to districts alongside institutionalisation of a mechanism for cross fertilisation through district participation, providing room for peer critique and forging joint solutions. In addition, a holistic tool was developed to guide the pooling of subnational reviews into the overall health sector performance assessment. This serves as a best case scenario that can be emulated by other countries.

### Weak mechanisms for follow-up

This was noted as a weakness in several countries. Frequently, implementation of recommendations from reviews and evaluation was reported as suboptimal by participants, and many felt that limited dissemination was one of the main obstacles to overcome. The most common modality of dissemination was the bulky and detailed review reports, which are not conducive for rapid internalisation. However, participants also gave examples of good practices. For example, in Malawi, the country develops a matrix for following up on recommendations with a timeframe and responsible officers. In Ghana and Uganda, an aide memoire between government actors and development partners indicates agreed upon actions for follow-up and is signed by all actors as a sign of commitment. One of the elements of good health policy dialogue is putting in place mechanisms for following up on recommendations [9].

### Lack of effective stakeholder participation

Institutionalising multi-sectoral collaboration and strengthening partnership frameworks remains a challenge in ensuring effective participation of all actors. Participants referred to inter-ministry relationships in terms of data sharing as ad hoc. Additionally, all countries noted that there were no routine mechanisms for collecting data from private-for-profit providers, meaning that a significant chunk of data for planning was excluded. However, in-line with the principle of mutual accountability, all health actors, including civil society, private-for-profit and private-not-for-profit actors, should all participate in assessing performance. Relatedly, there were challenges for donor programmes that supported implementation either through implementing partners or direct implementation by themselves. Concerns raised in this regard were poor sharing of data with the MoH since the implementing partners were only obliged to report to the donors. Likewise, donors were only sharing data with their head offices. A good practice here is the case of Rwanda, where all civil society organisations involved in the health sector are obliged to report to government by law. Such a practice can be emulated by other countries in a quest to ensure comprehensive performance assessment.

### Divergent political aspirations

This was in particular reference to decentralised health systems, which can further stress M&E systems by the proliferation of new districts, and also challenge the effectiveness of annual reviews and evaluations. For example, one country cited an example where subnational politicians disagreed with the review findings despite the fact that they had no contrary evidence. This further underlines questions of what indeed ‘evidence-based planning’ means.

## Discussion

Health sector performance assessments are only as useful as their ability to influence decision-making. Feeding the evidence that gets generated from them into regular country processes of sectoral monitoring, dialogue and policy modification is just a first step [19]. Health sector and programme performance assessments can provide rich contextual data that can inform health dialogue, planning and resource allocation. As such, efforts need to be made to maximise this opportunity.

This article raises several issues regarding the utility and relevance of annual reviews and evaluations for evidence-based health planning, in particular how they may be more greatly exploited to influence policy dialogue processes. There remain technical challenges, such as weak institutional capacities for M&E at national and subnational levels and weak data sources that need to be strengthened according to international standards and in a systematic way. Perhaps the most pertinent issue is the role of donor financing and donor priorities which can skew the focus of routine M&E country functions. The challenge of vertical programmes being better-resourced than other health areas is a persistent problem [20, 21]. Similarly, Chan et al. [22] noted that previous attempts have been fragmented, focussed on single disease information needs as opposed to strengthening health information systems in a systematic way. This raises two points. First, it requires strengthened governance at national level to ensure responsive investments of donor funds through evidence-based negotiation and clear articulation of locally-driven needs. Secondly, there is a need to share resources within the health sector, inputs such as technical officers, donor technical assistance and funding can be used to strengthen sectoral, and not only programmatic, M&E activities.

Lack of synchronisation between sectoral and programmatic planning cycles, and timeframes for conducting reviews and assessments, pose another challenge. Murray and Frenk [23] argue that coverage of public health programmes is an instrumental goal in itself that needs to be captured in the overall assessment of the health systems performance. In addressing this challenge, interventions are needed at both the global and national level. At the global level, actors need to commit to aligning their support to country strategies and planning cycles; while at the country level, strengthened MoH leadership would facilitate ensuring alignment of programme and sector strategic planning and implementation timeframes.

The inadequate time to undertake comprehensive and good quality performance assessments, as pointed out by the participants in the meeting, has also been highlighted by other scholars. Adam et al. [24] highlight inadequate timeframes, limited capacity and cost considerations as barriers to undertaking comprehensive assessments. Time constraints may be mitigated by improved access to data through strengthened routine health information systems [25], and more regularised partnerships with other government agencies collected data that is useful for assessing health sector performance.

Quite often, some recommendations for action from sector reviews remain unimplemented, especially if there is inadequate buy-in from the policy and decision makers. To get more recommendations implemented, closer interactive knowledge translation processes like policy dialogue, as highlighted by Panisset et al. [26], should be promoted. One way to do this is to set up knowledge translation platforms in countries to systematically use evidence in policymaking, a process that has already started in some of the countries [27].

The lack of effective participation of all health actors in data generation and use was also highlighted as a major issue. For instance, the exclusion of the private sector in sectoral M&E processes is a major gap, especially in some countries where the patronage of private health services is increasing. There are examples, however, of innovation in this regard. For example, in India, the provision of subsidies and capacity building resulted in increased reporting from private providers for tuberculosis services [28]. However, Lewin and Kaddar [29] caution that the weak regulatory capacity of government in low-income countries make scale-up of such models risky. The private-for-profit sector is a significant player in health service provision and efforts need to be made to streamline and enforce the collection and use of data through the routine health information systems. One option to address such issues would be to agree on a basic set of indicators that private-for-profit-providers could report on routinely.

There also exist political challenges in strengthening performance assessments for policy dialogue processes. This partly relates to improving multi-sectoral involvement in health sector annual reviews and evaluations; it also includes involvement of politicians. Blas et al. [30] highlight the fact that different viewpoints and understandings among stakeholders present a barrier to moving from rhetoric to action. Targeted dissemination using multiple channels has proven successful in allowing for focussed and clear messages to different audiences to stimulate action [31]. Blas et al. [30] reported similar findings, where some stakeholders took no action based on the evidence provided because they felt that the required action was outside their mandate and areas of influence. One option in addressing this challenge is seeking closer involvement of politicians through the entire process of analysing and interpreting data as opposed to sharing results at the very end. This has been shown to be beneficial in several ways, in that they understand the genesis of the recommendations, have more trust in the data and have been part of the process [32]. Jacobson et al. [33] developed a knowledge translation framework consisting of five domains: the user group, the issue, the research, the knowledge translation relationship, and dissemination strategies, with an emphasis on understanding the context of the user. Shroff et al. [34] recognise the critical importance of contextual and political factors and therefore advocate for consideration of leadership as an additional domain influencing the evidence-to-policy process for Jacobson’s framework to apply to low-income settings.

The totality of this reflection raises the question of whether it is possible or even desirable for country M&E plans to be standardised and harmonised. There are several inherent conflicts here. First, is the long-standing tension between standardisation, uniformity and control versus creativity, adaptation and organisational learning – top-down frameworks intrinsically work against the generative, organic quality of bottom-up processes [35, 36]. Furthermore, evidence has shown that the medium-term expenditure framework, which is designed to support operational planning and budgeting, in fact created challenges in its standardising approach. In a multi-country study of several sub-Saharan countries, Le Houerou and Talierco [37] found misalignments between higher-level resource allocation (e.g. at the level of the Ministry of Finance) and sectoral budgets, further complicating the difficulties of planning timeframes. Secondly, given the involvement of donor partners in programme funding and its influence on country M&E processes, it highlights the challenges of ill-defined harmonisation between donor procedures and country MoH procedures, which further leads to suboptimal local ownership [38]. However, we caution that, while the illustration of standardising within the medium-term expenditure framework is helpful, it is a fundamentally different process from standardising performance assessment.

## Conclusion

The question of what M&E is for in a country must be asked and answered clearly if the utility of these processes is to be maximised for informing policy dialogue and improving health sector performance. While it may be challenging and not always ideal to standardise planning and policy development processes for many reasons, among which is the shifting politics and donor funding cycles, arguments exist as to why standardising performance assessment is of great importance. It enables assessing performance over time and whether strategic plan objectives and policy aspirations are being met. Furthermore, it allows for comparisons across countries and subnational entities providing room for cross learning and replication of good lessons.

Identified challenges to standardising programmes and the health sector need to be overcome. There is a need for strengthened MoH leadership to ensure aligned health sector and programme strategies and plans, as well as responsive investments of donor funds in health information systems and skills building. Global efforts in this perspective, specifically the Health Data Collaborative initiative, which seeks to improve collective action to maximise the impact of the different partners’ investments in country health information systems, need to be exploited. Effective involvement of stakeholders right from data generation will increase ownership and confidence in the evidence and subsequently its use in policy dialogue and decision making. Follow-up mechanisms need to be instituted to ensure that findings from reviews inform action, are adaptively managed and ensure continued learning. In addition, health sector performance assessments need to be institutionalised as part of the accountability mechanism, must be planned for and the funding secured within annual budget and medium-term expenditure frameworks.

## Abbreviations

GHI: global health initiatives
M&E: monitoring and evaluation
MoH: Ministry of Health
